# The Prevalence of Addiction to Social Network Among Students in Iran and its Factors Related: A Study Conducted in 2020

**DOI:** 10.2174/1745017902117010170

**Published:** 2021-11-19

**Authors:** Hoorieh Rahiminia, Hamid Soori, Mahdi Jafari, Soheila Khodakarim

**Affiliations:** 1 Department of Epidemiology, School of Public Health and Safety, Shahid Beheshti University of Medical Sciences, Tehran, Iran; 2 Students’ Research Committee, School of Public Health and Safety, Shahid Beheshti University of Medical Sciences, Tehran, Iran; 3 Director of Safety Promotion and Injury Prevention Research Center, Shahid Beheshti University of Medical Sciences, Tehran, Iran; 4 Department of Clinical Psychology, School of Medicine, Shahid Beheshti University of Medical Sciences, Tehran, Iran; 5 Gastroenterology and Liver Diseases Research Center, Research Institute for Gastroenterology and Liver Diseases, Shahid Beheshti University of Medical Sciences, Tehran, Iran; 6 Department of Biostatistics, School of Medicine, Shiraz University of Medical Sciences, Shiraz, Iran

**Keywords:** Prevalence, Addiction, Social Networks, Students, Factors, Disciplines

## Abstract

**Background:**

In the present era, the prevalence of addiction to social networks has shown that many users, including students, are detaching from the real world. Therefore, this study aims to estimate the prevalence of addiction to the social networks among students in Iran and its related factors.

**Methods:**

This is a cross-sectional study conducted in 2020 on 1000 students in Shahid Beheshti University of Medical Sciences, Tehran, Iran. The data collection tool was a standardized questionnaire about addiction to the social networks distributed online among students. Statistical data were analyzed using Stata software version 14.

**Results:**

Generally, 462 students (44%) had addiction to social networks, of which 449 (90.04%) had slight addiction and 13 (9.96%) had severe addiction. The results showed that age (p = 0.001), type of disciplines in the treatment-related subgroup (p = 0.03) and using nerve medicine (p = 0.0001) had significant relationships with addiction to social networks.

**Conclusion:**

Considering that a high number of students in the present study are at high risk of addiction, it is suggested that educational programs should be implemented to improve the knowledge of parents and students for optimal use of social networks and raise awareness of the harmful consequences of addiction.

## 1 INTRODUCTION

Nowadays, different societies in the world, following the expansion of human relation and communication, have experienced the use of different modern mass communications like social networks. The virtual world has put the social and cultural life of communities under the influence of its positive or negative outcomes so that it sometimes helps strengthen and facilitate relationships and sometimes leads to unethical relationships, especially in adolescents [1, 2]. At present and especially during the COVID-19 pandemic, social networks have an important role in the students’ lives; therefore, students’ use of social networks should be screened to prohibit the addiction to the social networks in this age group [3].

Social networks such as Facebook, Twitter, WhatsApp, Telegram and Instagram, as the most important examples of online services, have become a place for the presence and familiarity of different segments of the society, especially the youth [4]. Addiction is defined as abnormal interaction that some people find with regard to some behaviors. While a person experiences a pleasant feeling as a consequence of addiction, the intensity of the individual’s dependence increases. In fact, it reinforces the behavioral pattern of addiction [5]. The addiction to social networks is motivated by the allocation of time and effort to use too much of these networks that disrupt other social activities, studies, jobs, interpersonal communication, psychological health, and well-being [4].

Young (1998) mentioned that having four symptoms such as having a feeling of preoccupation with the Internet, feeling a need to increase the time of use to satisfy the self, not being able to control its use, feeling agitated and restless while the Internet stops, using it to ease the emotion and find a way to escape from problems, and lying to others in order to hide the amount of its use are all necessary to be addicted to the social networks [6].

The number of Internet users in Iran (35.7%) is more than other countries in the Middle East, including Iraq (11.4%), Saudi Arabia (18.2%), United Arab Emirates (5.4%), and Jordan (5%). According to the results of this study, the number of Internet users in Iran has been increased from 3.8% to 68.5% between the years 2000 and 2016 [7, 8]. The prevalence of Internet addiction in students has been reported differently on the basis of studies. The addiction to Internet was 5.2% in students of Mashhad University of Medical Sciences in 2014. It was reported 33.8% for male students and 20% for female students at Zahedan University of Medical Sciences in 2016 and in Qum University of Medical Sciences, it was reported 28.7% in 2016 [9-11].

Along with all the advantages and disadvantages of social networks, addiction to these networks and the uncontrollable tendency of young people and adolescents to these networks without any cultural infrastructure can lead to disorders in all trends of life and their personalities and attitudes. Also, the prevalence of addiction to social networks has shown that many users, including students, allocate all their lives to these networks and thus fail to be involved in social activities and follow real life. Therefore, the present study aimed to investigate the prevalence of addiction to social networks among students in Iran and its related factors.

## 2 METHODS

### 2.1 Study Design and Setting

This research is a cross-sectional descriptive study conducted on students of Shahid Beheshti University of Medical Sciences, Tehran, Iran. It has 10130 students, 40 percentage of which live out of Tehran; therefore, these students could be a random light sample of Iranian students.

### 2.2 Participants

The study population consisted of 1000 students selected from 9 faculties at Shahid Beheshti University of Medical Sciences (No feedback was received from the other three faculties at the University) at BSc, MSc, MD, and Ph.D. levels. After registration of the plan and obtaining the required permission from the research department of Shahid Beheshti University of Medical Sciences that was approved by the University Ethics Committee (IR.SBMU.RETECH. REC.1399.032), the coordination with the faculties was performed. In each faculty, by referring to the office of academic affairs, necessary coordination was performed in order to provide the basis to collect data as an electronic questionnaire. Data collection through electronic questionnaires was conducted due to COVID-19 pandemic. First, the phone numbers of students’ representatives considering their major and entrance year were gathered from the office of academic affairs in each faculty. Then, the online questionnaires were sent to the representatives of classes through Press Line site. They were asked to provide the students with the questionnaire link through virtual groups of classes. Finally, 1000 questionnaires of 28 disciplines in 9 faculties were completed and returned using the self-administered method (Table **S1**).

### 2.2 Addiction to Social Networks Questionnaire

In this study, the Persian standardized version of the addiction to the social networks questionnaire was used [12]. This questionnaire was developed by Panayides *et al*. based on the Young Internet Addiction Test and included 20 Likert- type questions (very low =1, low =2, medium=3, high=4, very high=5) [13]. The higher score showed more addiction to the social networks in the students. The total score 20-49 showed normal user, the scores between 50 to 79 showed slight addiction, and the scores between 80 to 100 showed severe addiction to social networks.

### 2.3 Statistical Methods

The descriptive statistics showed the samples. For calculating the weighted prevalence rate, the distribution of the collected samples in terms of the type of the discipline and the number of students per discipline with distribution of population were compared and the sampling weights were calculated. First, the disciplines of the study were divided into two groups: treatment-related disciplines and others. The total number of disciplines in Shahid Beheshti University of Medical Sciences was 39 of which 17 disciplines were treatment-related disciplines (17/39=44%) and 22 disciplines were related to other disciplines (22/39=56%). The total number of the disciplines in our sample was 28 out of which 15 were treatment-related disciplines (15/28=54%) and 13 disciplines (13/28=46%) were related to other disciplines. Accordingly, weight1 (W1) was calculated from division of two numbers 44/54=0.81 for treatment-related disciplines and 56/46=1.21 for others (Table **S2**). In the next step, the number of students in each discipline was divided into the total number of students of the University (Medical n/N=2239/8433=27%), then the number of students of the sample in each discipline was divided to the total number of the students of the sample (Medical n/N =126/1000=13%). Weight2 (W2) was calculated from the division of these two amounts for each discipline(medical 27/13=2.07) (Table **S3**). Finally, the total weight (W3) gained by the multiplication of the first and second weights are listed in Table **1**.

In order to determine the factors related to addiction to social networks, weighted logistic regression with Stata software version 14 was used and sig < 0.05 was considered as a meaningful level.

## 3 RESULTS

The mean (standard deviation) of students’ age was 22.81 (4.66) years. Table **2** indicates that 658 (65.8%) were female, 895(89.5%) were single, 671 (67.1%) were BSc students and 118 (11.8%) were smokers (cigarette, hookahs or opioids).

Furthermore, of the 23 (2.3%) students who took antipsychotics, 47.83%, 30.43%, 13.04%, 4.35% and 4.35% reported that they had taken benzodiazepine (Clonazepam, Chlordiazepoxide, Alprazolam), selective serotonin reuptake inhibitor (SSRI: Fluoxetine, Asentra (Sertraline), Citalopram), tricyclic ani depressant (TCA: Clomipramine, Nortriptyline), serotonin norepinephrine reuptake inhibitor (SNRI: Duloxetine), and sleeping pills (Zolpidem), respectively.

Table **3** shows the distribution of students’ answers to the questions of the addiction to the social networks questionnaire. After recoding the students’ total score of the questionnaire, it was revealed that 462 students (44%) were addicted to the social networks of which 449 (90.04%) had slight addiction and 13 (9.96%) had severe addiction. Fig. (**1**) shows the prevalence of addiction to social networks in society.

Table **2** shows the association between the addiction to the social networks and demographic variables using simple and multiple weighted ordinal logistic regression method. To provide a brief explanation, the intercepts have not been reported. Our results show there is a significant statistical association between age and addiction to the social network (sig = 0.001). One-year increase in age would increase 8% of the chance of being in the group with higher level of addiction. There is a significant statistical association between the type of disciplines and the addiction to the social networks (sig = 0.03), and the chance of being in the group by which the higher level of the addiction in the treatment-related group is 49% more than the other disciplines group. Also, there is a significant statistical association between using nerve medicine and the addiction to the social network (sig=0.0001) and the chance of being in the group by which the higher level of the addiction by using these drugs was 46% more than the others.

There are no significant relationships between the addiction to the social networks and gender (sig = 0.47), marital status (sig = 0.06), level of education (sig >0.05) and smoking (sig=0.18).

## 4 DISCUSSION

According to this study, the overall prevalence of addiction to social networks was 462 (44%), and 538 (55%) were normal users.

In the Iranian population of the age group of 20 and older, the prevalence of social network addiction was 58% based on the study of Yarahmadi *et al*. (2020) by Young questionnaire [14]. Modara *et al*. (2017), in the meta-analysis study, have reported that the social networks addiction prevalence in Iran was 20% [15].

The reported prevalence of social networks or Internet addiction is different in Asian countries. In Iraq, the Internet addiction prevalence was 68% among the medical students at Baghdad University in 2019 detrmined by a study using Young questionnaire [16]. In India, the prevalence of Internet addiction was 3.7% in 2017, determined by a study using Young questionnaire [17]. A previous meta-analysis in Spain reported that the most frequently used scale was the IAT (Young Internet Addiction Test), and the prevalence of Internet addiction in teenagers ranged from 1.6% to 20.6% [18]. Singapore (2017), the rate of social networks addiction among the students by a researcher-made questionnaire was 29.5% [19]. The prevalence of the Internet addiction by Young questionnaire in Hong Kong 2012, Finland 2004 and Taiwan 2000 were 26.7%, 2.0% and 5.9%, respectively [20-22].

In addition, there is a large variation of the reported prevalence of social networks or Internet addiction in the other parts of the world. The Internet addiction prevalence (using Young questionnaire) in England 2013, Norway 2009, Italy 2006 and Australia 2001 was 3.2%, 1.0%, 5.4% and 4.0%, respectively [23-26].

As a consequence, conducting research on social networks addiction in students is necessary in different societies with different cultures and in the different times. It should be considered that the students are more at risk of severe addiction, especially during coronavirus pandemic because of spending more time on the internet and it can influence other aspects of their life.

Although the time of the data collection of this study was simultaneous with the starting of COVID-19 pandemic (23rd Aug, 2020 - 29th Oct, 2020), our study was not aimed at investigating the effects of this pandemic on the social network addiction (which could be performed if we had access to the data before the starting of this pandemic). As we emphasized, it was necessary to explain to the students that the questions regarding the time of using social networks are not applicable for any business activities they may have on social media. In addition, they were told not to consider the time they had to use the Internet due to coronavirus limitations like online classes and online shopping.

According to the results of this study, there was a significant relationship between the age of students and addiction to social networks. The results of this study indicated that the rate of addiction in younger age groups is more than other age groups, which can be due to the characteristics of the youth and adolescents and their greater use is due to different reasons in this period [17, 24, 27, 28].

The results of our study could not illustrate a significant relationship between the gender of students and the addiction to the social networks, which were consistent with the results of studies by Yarahmadi *et al*. [14], Fortson *et al*. [29], Kawabe *et al*. [30] but contradicted with studies by Ko *et al*. [31], Zboralski *et al*. [32], and Gorgich *et al*. [33].

There was a significant relationship between the addiction to the social networks and the type of disciplines. In this context, the results were consistent with Ahmadi *et al*. [34], Jafari *et al*. [35], and Hashemian *et al*. [36] but contradicted with other studies such as the study of Ozturk *et al*. [37]; this difference might be due to the multi-aspect use of the Internet and its different applications and objectives in all fields and disciplines.

Our results showed that there was no significant relationship between marital status and the addiction to the social networks, which was in accordance with some studies conducted on the students [9, 38]. Also, these results illustrated that there was no significant relationship between the level of education and the social networks, which was in accordance with the study of Imani *et al*. in 2018 [39].

This study has limitations, including the lack of honesty in filling out the questionnaire due to fear of personal information disclosure and recall bias. It could lead to underestimate the prevalence of the addiction to social networks among the students. Therefore, setting up similar studies in other universities is necessary.

## CONCLUSION

The addiction to the social networks among the Iranian young people, as a growing problem, needs to be considered by the experts and it could damage social life, scientific activities and carrier of the students. Therefore, developing and implementing strategies and educational programs for students, parents and university authorities are necessary to promote the proper use of social networks and prevent and reduce these harms.

## Figures and Tables

**Fig. (1):**
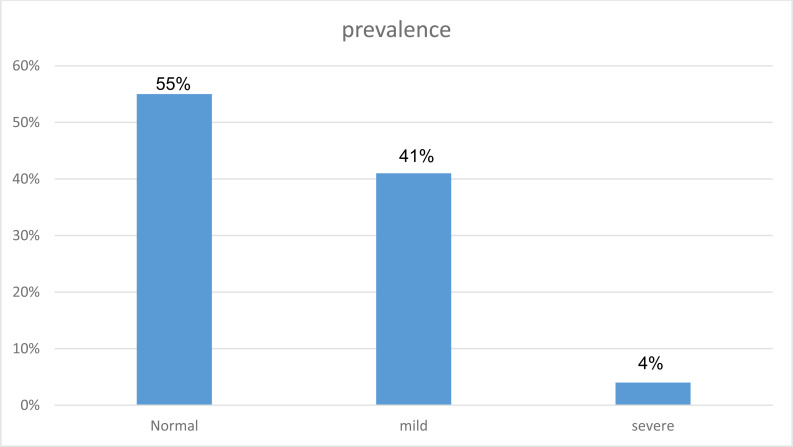
The weighted prevalence of the social networks addiction in students.

**Table 1:** Overall weight according to the sampling process by disciplines.

**-**	**Discipline**	**W1×W2=W3**
1	Medicine Doctor	2.07×0.81=1.67
2	Dentistry	1.60×0.81=1.30
3	Pharmacology	3.50×1.21=4.23
4	PhD Pharmacology	0.80×1.21=0.97
5	Nutrition	0.50×1.21=0.60
6	Food Technology	0.20×1.21=0.24
7	Laboratory sciences	0.50×0.81=0.40
8	Radiology	0.25×0.81=0.20
9	Radiotherapy	0.90×0.81=0.72
10	Medical informatic	0.40×1.21=0.48
11	Biostatistics	0.37×1.21=0.44
12	Audiology	1.00×0.81=0.81
13	Optometry	1.00×0.81=0.81
14	Physiotherapy	1.00×0.81=0.81
15	Occupational therapy	1.00×0.81=0.81
16	Public health	2.60×1.21=3.14
17	Occupational health	0.20×1.21=0.24
18	Environmental Health	0.14×1.21=0.16
19	Epidemiology	0.35×1.21=0.42
20	Industrial safety	1.00×1.21=1.21
21	Medical education	0.30×1.21=1.21
22	Surgical technologist	1.00×0.81=0.81
23	Anesthesiology	1.00×0.81=0.81
24	Midwifery	0.23×0.81=0.18
25	Nursing	1.40×0.81=1.13
26	Master of science nursing and midwifery	0.50×0.81=0.40
27	Dental protheses	1.50×0.81=1.21
28	Environmental health and safety management	0.80×1.21=0.96

w1: Discipline weight. w2: Student weight in each discipline.

**Table 2:** Relationship between social network addiction and the demographic variables using Weighted Ordinal Logistic Regression.

Demographic Variables	Level	Number	Raw Odds Ratio (CI 95%)	Sig.	Adjusted Odds Ratio (CI 95%)	Sig.
Age	-	-	0.92 (0.87-0.96)	0.001	0.90 (0.85-0.96)	0.002
Gender	male	342	1.13 (0.80-1.60)	0.47	1.08 (0.74-1.57)	0.66
	female	658	-	-	-	-
Marital status	married	105	0.46 (0.20- 1.04)	0.06	0.76 (0.34- 1.66)	0.49
	single	895	-	-	-	-
Discipline	related to treatment	488	1.49(1.02-2.18)	0.03	1.51 (1.01-2.24)	0.04
	others	512	-	-	-	-
Educational level	Ph.D	70	0.61 (0.27-1.36)	0.23	0.76 (0.51-1.14)	0.19
	Medicine Doctor+ Master of science	259	0.83 (0.57-1.20)	0.34	0.88 (0.34-2.25)	0.80
	Bachelor	671	-	-	-	-
Smoker	Yes	118	1.52(0.82-2.81)	0.18	1.28(0.75-2.20)	0.35
	No	882	-	-	-	-
Sedatives drugs	Yes No	23 977	1.46(5.13-4.13) -	0.0001 -	1.13(3.31-3.84) -	0.0001 -

**Table 3:** The distribution of students’ response to the questionnaire of “addiction to the social networks” questionnaire.

	Questions	Very Low	Low	Medium	High	Very High
1	How much longer do you spend on social networks than you do?	75(7.5%)	155(15.5%)	377(37.7%)	304(30.4%)	89(8.9%)
2	How often do you ignore family members for staying online?	169(16.9%)	327(32.7%)	312(31.2%)	145(14.5%)	47(4.7%)
3	How much do you prefer social networks to family?	241(24.1%)	317(31.7%)	276(26.7%)	132(13.2%)	34(3.4%)
4	How much do you communicate with other users through social networks?	132(13.2%)	196(19.6%)	320(32.0%)	276(27.6%)	76(7.6%)
5	How dissatisfied are others with you because you are online?	299(29.9%)	358(35.8%)	205(20.5%)	110(11.0%)	28(2.8%)
6	How much have your college grades and work dropped due to social networks?	378(37.8%)	330(33.0%)	179(17.9%)	79(7.9%)	25(2.5%)
7	How often do you check social networks before your other essentials?	155(15.5%)	234(23.4%)	275(27.5%)	261(26.1%)	75(7.5%)
8	How much the efficiency of your daily useful work damaged by social networks?	175(17.5%)	331(33.1%)	273(27.3%)	163(16.3%)	58(5.8%)
9	When you asked what you do online, how defensive or secretive you get ?	298(29.8%)	411(41.1%)	215(21.5%)	59(5.9%)	17(1.7%)
10	How much do you neutralize annoying thoughts in life with soothing thoughts on social networks?	176(17.6%)	256(25.6%)	329(32.9%)	189(18.9%)	50(5.0%)
11	How much do you feel you have the ability to control life affairs when you are on social networks?	176(17.6%)	341(34.1%)	345(34.5%)	124(12.4%)	14(1.4%)
12	How boring and empty do you think life is without social networks?	187(18.7%)	217(21.7%)	262(26.2%)	228(22.8%)	106(10.6%)
13	How much do you get moan, scream or get angry when you are online and someone disturb you?	390(39.0%)	334(33.4%)	185(18.5%)	76(7.6%)	15(1.5%)
14	How much sleep do you lose because you are on social networks late at night?	250(25.0%)	235(23.5%)	233(23.3%)	197(19.7%)	85(8.5%)
15	How much do you think you get distracted when you are offline?	387(38.7%)	313(31.3%)	162(16.2%)	101(10.1%)	37(3.7%)
16	How often do you use this phrase when you are online: only some minutes needed then I will come	217(21.7%)	263(26.3%)	244(24.4%)	211(21.1%)	65(6.5%)
17	How much have you tried to reduce your online presence and failed?	224(22.4%)	315(31.5%)	256(25.6%)	155(15.5%)	50(5.0%)
18	How much do you try to hide your online presence from others?	391(39.1%)	320(32.0%)	166(16.6%)	97(9.7%)	26(2.6%)
19	How much do you prefer being online to going out with others?	519(51.9%)	251(25.1%)	140(14.0%)	58(5.8%)	32(3.2%)
20	How much do you feel uncomfortable and angry when you are offline which can be fixed by getting online?	396(39.6%)	317(31.7%)	182(18.2%)	75(7.5%)	30(3.0%)

## Data Availability

The data that supports the findings of this study are available from the corresponding author [S.K.] upon reasonable request.
