# Supplementary material for: The Prevalence of Addiction to Social Network Among Students in Iran and its Factors Related: A Study Conducted in 2020
Source: Clin Pract Epidemiol Ment Health. 2021 Nov 19;17:170–6. doi: 10.2174/1745017902117010170 (PMC8719277; doi:10.2174/1745017902117010170)
Supplement: Supplementary file 1 [file CPEMH-17-170_SD1.pdf]

1745-0179/21 Send Orders for Reprints to reprints@benthamscience.net

Clinical Practice & Epidemiology in Mental Health

Content list available at: https://clinical-practice-and-epidemiology-in-mental-health.com

The Prevalence of Addiction to Social Network Among Students in Iran and its Factors Related: A Study Conducted in 2020

Hoorieh Rahiminia[1,2], Hamid Soori[1,3], Mahdi Jafari[4,5] and Soheila Khodakarim[1,6,*]

[1]*Department of Epidemiology, School of Public Health and Safety, Shahid Beheshti University of Medical Sciences, Tehran, Iran*
[2]*Students' Research Committee, School of Public Health and Safety, Shahid Beheshti University of Medical Sciences, Tehran, Iran*
[3]*Director of Safety Promotion and Injury Prevention Research Center, Shahid Beheshti University of Medical Sciences, Tehran, Iran*
[4]*Department of Clinical Psychology, School of Medicine, Shahid Beheshti University of Medical Sciences, Tehran, Iran*
[5]*Gastroenterology and Liver Diseases Research Center, Research Institute for Gastroenterology and Liver Diseases, Shahid Beheshti University of Medical Sciences, Tehran, Iran*
[6]*Department of Biostatistics, School of Medicine, Shiraz University of Medical Sciences, Shiraz, Iran*

Table S1. The frequency distribution of students of Shahid Beheshti University of Medical.

Faculty		Number
**Faculties were in the study**	Medicine Doctor	2239
	Dentistry	684
	Public health and safety	1302
	Allied medical sciences	399
	Rehabilitation	700
	Nursing and Midwifery	1659
	Pharmacology	710
	Nutrition	501
	Medical Education and Management	150
	total	8344
**Others Faculties**	Traditional medicine	442
	Advanced technologies in medicine	388
	VARAMIN Health Higher Education Complex	956
	total	1786
**total**	-	**10130**

Table S2. Classification of fields into two groups and their weights in Shahid Beheshti University of Medical Sciences in the present sample.

Treatment-related disciplines	Medicine doctor, Dentistry, Dental Prosthesis, Surgical technologist, Midwifery, Nursing, Master of science nursing, Anesthesiology, Laboratory sciences, Radiology, Radiotherapy, Audiology, Optometry, Physiotherapy, Occupational therapy
Other disciplines	Pharmacology, PhD Pharmacology, Medical education, Industrial safety, Nutrition, Food technology, Biostatistics, Medical informatics, Environmental health and safety management, Public health, Occupational health, Environmental health, Epidemiology

(Contd.....

Fraction in sample	Treatment-related disciplines	15/28~0.54
	Other disciplines	13/28~0.46
Fraction in Population	Treatment-related disciplines	17/39~0.44
	Other disciplines	22/39~0.56
Weight 1	Treatment-related disciplines	44/54~0.81
	Other disciplines	56/46~1.21

Table S3. The number and weights of students of each discipline in Shahid Beheshti University of Medical Sciences.

	Discipline	Population (N/n)	sample (N/n)	Weight 2 (P/S)
1	Medicine Doctor	2239/8433=0.265 (27%)	126/1000=0.126= (13%)	27/13=2.07
2	Dentistry	684/8433=0.08(8%)	58/1000=0.05= (5%)	8/5=1.6
3	Pharmacology	640/8433=0.07(7%)	25/1000=0.02(2%)	7/2=3.5
4	Ph.D Pharmacology	70/8433=0.008(0.8%)	10/1000=0.01= (1%)	0.8/1=0.8
5	Nutrition	311/8433=0.03(3%)	65/1000=0.06= (6%)	3/6=0.5
6	Food Technology	190/8433=0.02(2%)	103/1000=0.103= (10%)	2/10=0.2
7	Laboratory sciences	135/8433=0.01(1%)	24/1000=0.02= (2%)	1/2=0.5
8	Radiology	134/8433=0.01(1%)	41/1000=0.04(4%)	1/4=0.25
9	Radiotherapy	80/8433=0.009(0.9%)	10/1000=0.01= (1%)	0.9/1=0.9
10	Medical informatics	21/8433=0.002(0.2%)	5/1000=0.005(0.5%)	0.2/0.5=0.4
11	Biostatistics	29/8433=0.003(0.3%)	8/1000=0.008(0.8%)	0.3/0.8=0.37
12	Audiology	124/8433=0.01(1%)	11/1000=0.01(1%)	1/1=1
13	Optometry	174/8433=0.02(2%)	24/1000=0.02= (2%)	2/2=1
14	Physiotherapy	260/8433=0.03(3%)	31/1000=0.03(3%)	3/3=1
15	Occupational therapy	142/8433=0.01(1%)	12/1000=0.012(1%)	1/1=1
16	Public health	700/8433=0.08(8%)	36/1000=0.03(3%)	8/3=2.6
17	Occupational health	242/8433=0.02(2%)	103/1000=0.103= (10%)	2/10=0.2
18	Environmental Health	127/8433=0.01(1%)	75/1000=0.07(7%)	1/7=0.14
19	Epidemiology	60/8433=0.007(0.7%)	25/1000=0.02= (2%)	0.7/2=0.35
20	Industrial safety	88/8433=0.01(1%)	16/1000=0.01(1%)	1/1=1
21	Medical education	30/8433=0.003(0.3%)	12/1000=0.012(1%)	0.3/1=0.3
22	Surgical technologist	139/8433=0.01(1%)	10/1000=0.01= (1%)	1/1=1
23	Anesthesiology	162/8433=0.01(1%)	13/1000=0.013(1%)	1/1=1
24	Midwifery	60/8433=0.007(0.7%)	34/1000=0.03= (3%)	0.7/3=0.23
25	Nursing	1131/8433=0.13(13%)	97/1000=0.09(9%)	13/9=1.4
26	Master of science nursing and midwifery	167/8433=0.01(1%)	21/1000=0.02= (2%	1/2=0.5
27	Dental prostheses	256/8433=0.03(3%)	24/1000=0.02(2%)	3/2=1.5
28	Environmental health and safety management	40/8433=0.004(0.4%)	5/1000=0.005(0.5%)	0.4/0.5=0.8

© 2021 Rahiminia *et al*.

This is an open access article distributed under the terms of the Creative Commons Attribution 4.0 International Public License (CC-BY 4.0), a copy of which is available at: https://creativecommons.org/licenses/by/4.0/legalcode. This license permits unrestricted use, distribution, and reproduction in any medium, provided the original author and source are credited.
